# (Dis)concordance of comorbidity data and cancer status across administrative datasets, medical charts, and self-reports

**DOI:** 10.1186/s12913-020-05713-5

**Published:** 2020-09-11

**Authors:** A. Sheriffdeen, J. L. Millar, C. Martin, M. Evans, G. Tikellis, S. M. Evans

**Affiliations:** 1 Department of Epidemiology & Preventive Medicine, Monash University, Melbourne, Australia; 2 William Buckland Radiotherapy Centre, The Alfred, Melbourne, Australia

**Keywords:** Prostate cancer, Concordance, Self-reports, Comorbidities

## Abstract

**Background:**

Benchmarking outcomes across settings commonly requires risk-adjustment for co-morbidities that must be derived from extant sources that were designed for other purposes. A question arises as to the extent to which differing available sources for health data will be concordant when inferring the type and severity of co-morbidities, how close are these to the “truth”. We studied the level of concordance for same-patient comorbidity data extracted from administrative data (coded from International Classification of Diseases, Australian modification,10th edition [ICD-10 AM]), from the medical chart audit, and data self-reported by men with prostate cancer who had undergone a radical prostatectomy.

**Methods:**

We included six hospitals (5 public and 1 private) contributing to the Prostate Cancer Outcomes Registry-Victoria (PCOR-Vic) in the study. Eligible patients from the PCOR-Vic underwent a radical prostatectomy between January 2017 and April 2018.Health Information Manager’s in each hospital, provided each patient’s associated administrative ICD-10 AM comorbidity codes. Medical charts were reviewed to extract comorbidity data. The self-reported comorbidity questionnaire (SCQ) was distributed through PCOR-Vic to eligible men.

**Results:**

The percentage agreement between the administrative data, medical charts and self-reports ranged from 92 to 99% in the 122 patients from the 217 eligible participants who responded to the questionnaire. The presence of comorbidities showed a poor level of agreement between data sources.

**Conclusion:**

Relying on a single data source to generate comorbidity indices for risk-modelling purposes may fail to capture the reality of a patient’s disease profile. There does not appear to be a ‘gold-standard’ data source for the collection of data on comorbidities.

## Background

Prostate cancer (CaP) is the most common non-skin cancer in men worldwide [1]. In Australia, it represents the second leading cause of cancer-related mortality in males [2]. Optimal disease management prevents progression of the cancer and preserves quality of life through avoidance of unnecessary treatment. The need to monitor the health and wellbeing of men with CaP has been recognised by physicians and healthcare workers, leading to the establishment of clinical quality registries. A clinical registry “collect(s) uniform data to evaluate specific outcomes for a population defined by a specific disease, condition or exposure that serves one or more predefined scientific, clinical or policy purpose” [3].

The Prostate Cancer Outcome Registry-Victoria (PCOR-Vic) was developed in 2009 as a clinical quality registry, to measure and report on quality of care, using benchmarking of performance at a clinician and hospital level. Benchmarking is one of the most effective strategies for quality improvement, as it provides useful information to medical professionals on where to improve clinical practice [4]. PCOR-Vic collects data on aspects relating to the diagnosis, treatment, quality of life and clinical outcomes of men diagnosed with CaP [5]. Quality of care is examined using specific quality of care indicators (see Supplementary Table 1); selected after a literature review and a prioritisation process (modified Delphi [6]) which considered the importance and feasibility of each proposed quality indicator. Indicators were categorised according to whether they assessed structures, processes or outcomes of care [7]. Both clinical (e.g. positive surgical margins and 90-day prostate cancer specific mortality (PCSM) following treatment) and patient-reported outcomes (urinary, bowel and sexual quality of life) are reported.

Positive surgical margins (PSMs) and 90-day mortality are currently risk-adjusted using the National Comprehensive Cancer Network (NCCN) risk model, which considers the clinical stage prior to treatment, including the prostate specific antigen (PSA) level and the Gleason score at biopsy, but not patient comorbidities [8]. Patient-reported outcomes are stratified according to the treatment modalities (surgery, radiotherapy, androgen deprivation therapy (ADT), active surveillance/watchful waiting). If comorbidities are found to be independently associated with outcomes such as PSMs and PCSM, it may be appropriate to include them in the risk models used to generate benchmark reports for hospitals and clinicians. Additionally, the inclusion of comorbidities in modelling may be used for pre-operative risk stratification, provision of information during the informed consent process with patients and their families, as well as in decision-making regarding the suitability for patients to undergo surgery. Previous studies have suggested how obesity, a common comorbidity, is correlated with PSMs and PCSM [9, 10].

However, prior to adjusting for comorbidities in the risk models, it is essential to understand the extent to which they are accurately captured in the data source from which they will be extracted. Comorbidity data may be derived from International Classification of Diseases (ICD) codes, manually abstracted from medical charts or collected directly from patients. It is unclear as to whether there would be concordance between each of these data sources and what would be considered the ‘gold standard’. Clinical notes might be incomplete, the patient may not be informed of all comorbidities and the coding system may not document all comorbidities.

The aim of this project was to examine the completeness and agreement of comorbidity data and cancer status obtained from three data sources: ICD coded administrative data, medical charts and self-reports in men contributing to PCOR-Vic who have had a radical prostatectomy following a diagnosis of prostate cancer.

## Methods

### Study design

A retrospective cohort study design was employed for this project. Men contributing to PCOR-Vic who had undergone a radical prostatectomy between January 2017 and April 2018 at one of six convenient hospitals were eligible to participate in this study. The five public hospitals included four metropolitan and one regional hospital, while the private hospital was located in metropolitan Melbourne. These six major health care facilities were chosen to maximise the sample size at each site.

A patient information and consent form, along with the questionnaire, were sent to eligible men.

### Rationale behind chosen diseases

The diseases analysed in this study are based on the conditions included within the Charlson Comorbidity Index (CCI). The CCI is a universally employed index used to quantify comorbidity in ill patients, with weightings being assigned to each comorbidity depending on severity (see Supplementary Table 2) [11]. It captures 19 comorbidities. The questionnaire administered to patients in this study was developed from this index [12].

For the purposes of inter-data comparison, a modified weighted CCI (mCCI) was developed which contained only the eight variables present in both the CCI and the self-reported comorbidity questionnaire (SCQ). As the CCI weighted liver disease, diabetes, and cancer based on their severity (moderate or severe), two mCCI scores were computed for each patient to factor in the possibility that a patient had a milder or more severe version of the condition, as the severity of each of these three was not captured in the self-reported comorbidity survey.

### Comorbidity data sources

Three data sources were used to compare comorbidities:

1. The mCCI was calculated from the administrative ICD-10 AM codes, using work developed by Sundararajan et al. [13]
2. The SCQ made available to men was to be completed either on paper, online or via telephone (see Supplementary Table 3) [12]. The questionnaire included eight comorbidities captured by the CCI and five additional comorbidities: hypertension, depression, osteoarthritis, back pain and ‘anaemia/other blood disease’. Out of the six chosen sites, one site failed to provide administrative information pertaining to these five conditions.
3. A medical chart audit was undertaken to capture CCI comorbidities reported in both the CCI and the SCQ, identified during the hospital admission related to the radical prostatectomy. For patients treated in the private hospital, the medical audit was undertaken using medical charts held in consulting rooms.

### Statistical analysis

Characteristics of the respondents are reported as medians and interquartile ranges due to the skewed distribution or ordinal nature of variables such as age, PSA levels, Gleason score. The Index of Relative Socio-economic Advantage and Disadvantage (IRSAD) was used to assess socio-economic status. An IRSAD score of 0 represented the greatest societal disadvantage and a score of ten represented the greatest societal advantage [14]. Statistical significance was defined as a *p*-value < 0.05.

Concordance was calculated using percentage agreement and the kappa statistic. The level of agreement for the kappa statistics was defined as follows: “poor” agreement was defined as a score less than zero, “slight” agreement was defined as a score of 0 to 0.20, “fair” agreement was defined as a score of 0.21 to 0.40, “moderate” agreement was defined as a score of 0.41 to 0.60, “substantial” agreement was defined as a score of 0.61 to 0.80 and “almost perfect” agreement was defined as a score of 0.81 to 1.00 [15].

Stata 15 (StataCorp, College Station, Texas) was used for data analysis.

The ethics application for this study was approved by the Monash University Human Research Ethics Committee (HREC/18/MonH/62). Governance approvals were obtained from each hospital.

## Results

**Fig. 1:**
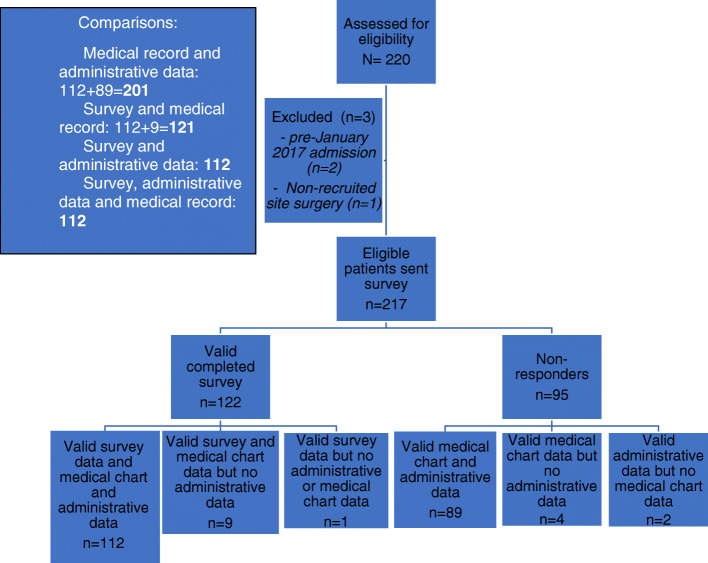
Recruitment frame

The recruitment frame is described in Fig. 1. Table 1 describes the characteristics of the cohort contributing to this analysis. The median age of the 217 participants was 66 years (IQR, 9 years). The median PSA level and Gleason score at diagnosis was 6.9 ng/mL (IQR, 4.7 ng/mL) and 7 (IQR, 1.), respectively. The majority of patients had an IRSAD score ≥ 8 indicating they resided in a more advantageous socio-economic area. Most patients in this study were classified in the NCCN intermediate risk group for disease progression.

**Table 1:** Characteristics of study sample (*N* = 217)

Characteristic (***N*** = 217)	n	Median^**a**^ (IQR)
Age	217	66.0 (9.0)
PSA level at diagnosis	200	6.9 (4.7)
Gleason sum score at diagnosis	211	7 (1.)
NCCN risk of disease progression group		
Very Low	25	–
Low	22	–
Intermediate	120	–
High	40	–
Very High	2	–
Metastatic	3	–
Index of Relative Social Advantage and Disadvantage (IRSAD) group		
0–1	18	–
2–3	20	–
4–5	46	–
6–7	48	–
≥ 8	85	–
Modified Charlson Comorbidity Index (mCCI)		
mCCI – administrative data	203	2 (0)
mCCI- medical charts	214	2 (0)
mCCI- self reported [mild]	122	0 (0)
mCCI- self reported [severe]	122	0 (0)
mCCI- self reported [mild, cancer assumed]	122	2 (0)
mCCI- self reported [severe, cancer assumed]	122	6 (0)

^a^Medians (interquartile range –IQR) reported due to skewed distribution of variables

The median mCCI score based on the administrative data and medical chart audit was 2 (IQR, 0). The median mCCI score based on the patient self-reports’ mild and severe assumptions were 0 (IQR, 0), due to the large proportion of patients not self-reporting any comorbidity. With the assumption that all self-reported patients had cancer, the median mCCI score based on the patient self-reports’ mild and severe assumptions was 2 (IQR, 0) and 6 (IQR, 0), respectively (Table 1). All further analysis report only on comparisons across the three data sources using the mCCI. CCI scores were not considered for comparison as the SCQ did not collect information on five Charlson conditions. The median comorbidity count, for the administrative data, self-reports and medical chart review were 1 (IQR, 0), 1 (IQR, 1), and 2 (IQR, 2) respectively.

**Fig. 2:**
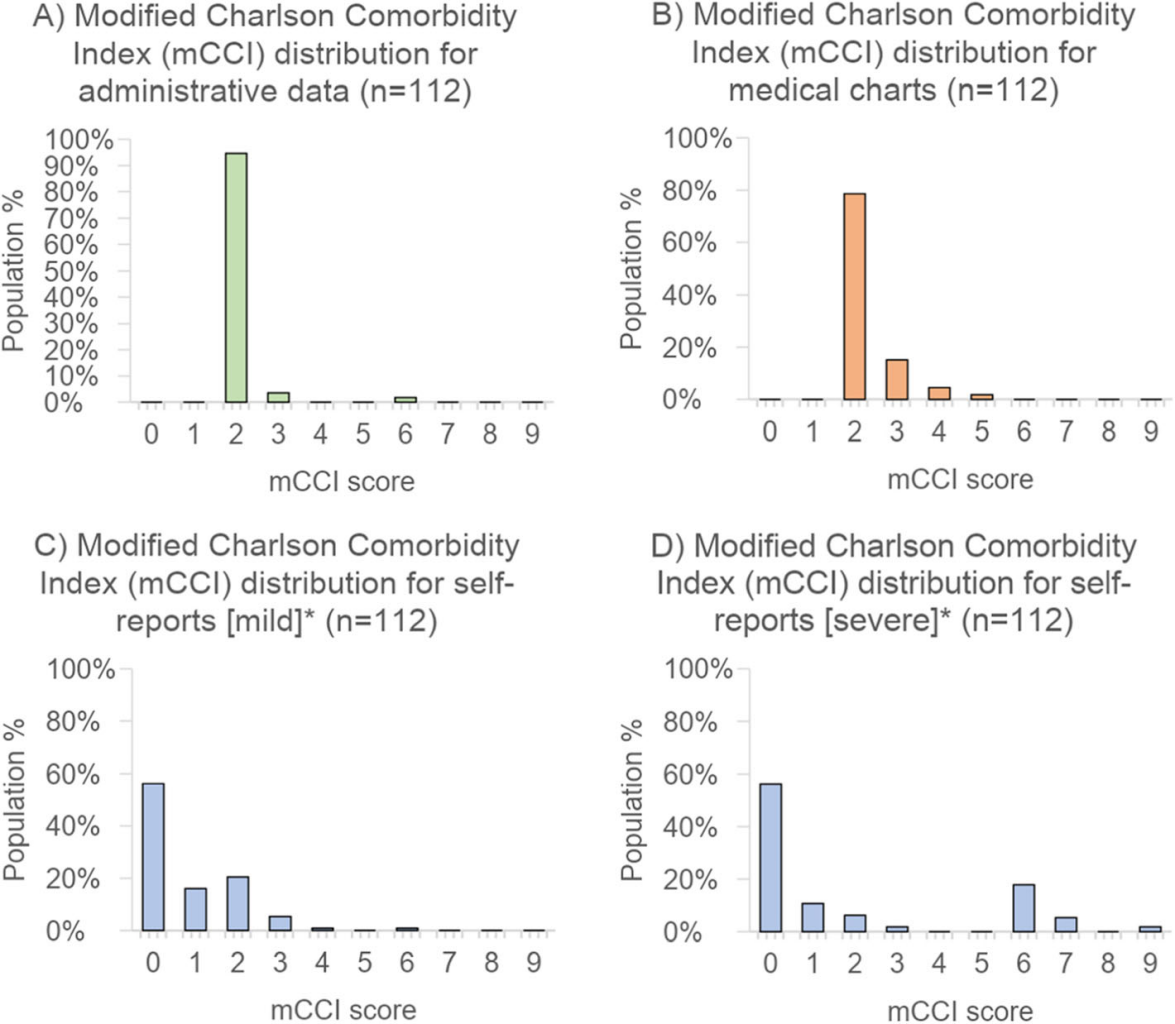
Distribution of comorbidity scores according to administrative data, medical record review and self-reports using the Modified Charlson Comorbidity Index. *Self reports were computed using two scenarios-mild or severe- for each of three comorbidities weighted by the mCCI (liver disease, cancer and diabetes), as this was not captured in self-reports

Figure 2a-d depicts the distribution of comorbidities for the 112 patients for whom data was available in all three sources. All distributions follow a discrete number scale due to the mCCI being a non-continuous comorbidity index. Based on administrative and medical chart distributions (Fig. 2a and b), no patients had a CCI score of 0 or 1, as cancer was recorded for each patient, and this carries a weighting of 2 in the CCI. In 22.3% of patients, the administrative dataset had a mCCI score ≥ 3, in contrast to only 5.7% of patients in the medical charts. The patient self-report CCI distributions indicate that 56.3% of patients had CCI of zero (no self-reported comorbidities) (Fig. 2c-d). In 0.9% of patients, the CCI score was ≥6 based on the mild comorbidity assumption (Fig. 2c), with this figure increasing to 25% for the severe comorbidity assumption (Fig. 2d). The patient self-report distributions were notably more spread out compared to the administrative dataset and medical chart-based distributions.

Site 1 did not provide the complete administrative information for the patients’ comorbidities.

### Medical charts vs administrative datasets

Table 2 reports on the level of statistical concordance between the medical chart and administrative datasets. In total 201 cases were compared; 392 comorbidities were identified through audit of medical chart and 310 through administrative data. Three out of the thirteen analysed conditions were unable to have a reporting statistic computed due to the prevalence of the condition being either 0% or 100% in both of the compared datasets. Of the remaining comorbidities, the kappa statistic reported “poor” concordance between the medical chart and administrative data other than for diabetes, which had a “substantial agreement”.

**Table 2:** Agreement for presence of selected comorbidities between medical chart and administrative data

	Comorbidity	Medical chart data n, (%)	Administrative data n, (%)	Percentage agreement %, (95% CI)	κ statistic (95% CI)
**Data available for all six study sites (*n*** **= 201)**	**Myocardial infarction** **Congestive heart failure**	6 (3.0)	0 (0)	97 (95–99)	0 (0–0)
	**Diabetes without chronic complications** **Diabetes with chronic complications**	21 (10.5)	17 (8.5)	94 (90–97)	0.61 (0.42–0.81)
	**Mild liver disease** **Moderate or severe liver disease**	3 (1.5)	1 (0)	99 (97–100)	0 (0–0)
	**Any malignancy**^**a**^ **Metastatic solid tumour**	201 (100)	201 (100)	NC	NC
	**Rheumatic disease**^**b**^	4 (2.0)	(0)	98 (96–100)	(0–0)
	**Ulcer or stomach disease**	3 (1.5)	0 (0)	99 (97–100)	0 (0–0)
	**Renal disease**	4 (2.0)	1 (0.5)	98 (95–100)	−0.01 (−0.02–0.00)
	**Chronic pulmonary disease**	26 (12.9)	0 (0)	88 (83–92)	0 (0–0)
	**Hemiplegia**	0 (0)	0 (0)	NC	NC
	**AIDS/HIV**	0 (0)	0 (0)	NC	NC
	**Peripheral vascular disease**	1 (0.5)	0 (0)	100 (99–100)	0 (0–0)
	**Dementia**	0 (0)	0 (0)	NC	NC
	**Cerebrovascular disease**	5 (2.5)	0 (0)	98 (96–100)	0 (0–0)
**Data available for all sites except site 1 (*n*** **= 172)**	**High blood pressure**	81 (47.1)	72 (41.9)	87 (81–92)	0.73 (0.63–0.83)
	**Anaemia or other blood disease**	7 (4.1)	9 (5.2)	93 (89–97)	0.21 (−0.08–0.51)
	**Depression**	6 (3.5)	0 (0)	97 (94–99)	0 (0–0)
	**Back pain**	7 (4.1)	0 (0)	96 (93–99)	0 (0–0)
	**Osteoarthritis/ degenerative arthritis**	17 (9.9)	9 (5.2)	93 (89–97)	0.50 (0.26–0.75)
	**Total count**	392	310		

*NC* not calculable (prevalence was either 0% or 100%)

^a^includes leukaemia and lymphoma

^b^same as connective tissue disorders

### Medical chart vs self-reports

Table 3 compares comorbidities captured by medical chart and patient self-report data using the SCQ. Of the 121 cases present in both cohorts, medical charts detected 237 comorbidities, while patients reported 203 comorbidities. Based on the kappa score, concordance ranged from “poor” to “substantial”, with the best agreement being attributed to diabetes with a kappa score of 0.71. Cancer was classified to have extremely “poor” concordance, with negative concordance scores being cited.

**Table 3:** Agreement for presence of selected comorbidities between medical chart and self-reported comorbidity questionnaire (SCQ) (*n* = 121)

Comorbidity	Medical chart Present, n (%)	SCQ data Present, n (%)	Percentage agreement %, (95% CI)	κ statistic (95% CI)
**Heart disease**	2 (1.6)	9 (7.4)	93 (88–97)	0.16 (−0.15–0.46)
**Diabetes**	9 (7.4)	10 (8.3)	96 (92–99)	0.71 (0.47–0.96)
**Liver disease**	1 (0.8)	1 (0.8)	98 (96–100)	−0.01 (− 0.02–0.00)
**Cancer**	121 (100)	31 (25.6)	26 (18–34)	0 (0–0)
**Rheumatic disease**^**a**^	2 (1.7)	7 (5.8)	94 (90–98)	0.20 (−0.16–0.57)
**Ulcer or stomach disease**	1 (0.8)	4 (3.3)	96 (92–99)	−0.01 (− 0.04–0.01)
**Renal disease**	3 (2.5)	1 (0.8)	97 (93–100)	−0.01 (− 0.03–0.01)
**Chronic pulmonary disease**	12 (9.9)	5 (4.1)	91 (86–96)	0.31 (0.01–0.61)
**High blood pressure**	56 (46.3)	51 (42.2)	84 (78–91)	0.68 (0.55–0.81)
**Anaemia or other blood disease**	6 (5.0)	3 (2.5)	96 (92–99)	0.43 (0.01–0.84)
**Depression**	6 (5.0)	17 (14.1)	88 (82–94)	0.30 (0.04–0.55)
**Back pain**	4 (3.3)	38 (31.4)	72 (64–80)	0.14 (0.01–0.27)
**Osteoarthritis/degenerative arthritis**	14 (11.6)	26 (21.5)	75 (67–83)	0.12 (−0.08–0.31)
**Total count**	237	203		

^a^same as connective tissue disorders

### Administrative data vs self-reports

Table 4 summarises the level of concordance between administrative data and patient self-report data using the SCQ. Only eight conditions were comparable. Of the 112 cases compared between these two datasets, administrative data identified 174 comorbidities while 165 were self-reported by patients. Due to the relatively small number of cases reported for each comorbidity in both datasets, the kappa score reported “poor” agreement between the administrative data and self-reports, with the exception of diabetes which had “almost perfect” agreement.

**Table 4:** Agreement for presence of selected comorbidities between administrative data

	Comorbidity	Administrative data Present, n (%)	SCQ data Present, n (%)	Percentage agreement %, (95%CI)	κ statistic (95% CI)
**Data available for all six study sites (*n*=112)**	**Heart disease**	0 (0)	9 (8.0)	92 (87-97)	0 (0-0)
	**Diabetes**	7 (6.3)	10 (8.9)	97 (94-100)	0.81 (0.60-1.00)
	**Liver disease**	0 (0)	1 (0.9)	99 (97-100)	0 (0-0)
	**Cancer**	112 (100)	27 (24.1)	24 (16-32)	0 (0-0)
	**Rheumatic disease**^**a**^	0 (0)	7 (6.3)	94 (89-98)	0 (0-0)
	**Ulcer or stomach disease**	0 (0)	4 (3.6)	96 (93-100)	0 (0-0)
	**Renal disease**	1 (0.9)	1 (0.9)	98 (96-100)	-0.01 (-0.02-0.00)
	**Chronic pulmonary disease**	0 (0)	5 (4.5)	96 (92-99)	0 (0-0)
**Data available for all sites except site 1 (*n*=98)**	**High blood pressure**	42 (42.9)	40 (40.8)	88 (81-94)	0.75 (0.61-0.88)
	**Anaemia or other blood disease**	6 (6.1)	2 (2.0)	96 (92-100)	0.48 (0.05-0.92)
	**Depression**	0 (0)	11 (11.2)	89 (82-95)	0 (0-0)
	**Back pain**	0 (3)	26 (26.5)	73 (65-82)	0 (0-0)
	**Osteoarthritis/degenerative arthritis**	6 (6.1)	22 (22.4)	78 (69-86)	0.13 (-0.07-0.33)
	**Total count**	174	165		

^a^same as connective tissue disorders

### Comparison across the three data sources

Table 5 provides a summary of the concordance of comorbidity data across the three data sources. Apart from diabetes, the Kappa statistic suggested a poor to slight agreement for seven of the eight comorbidities. Cancer had the lowest level of agreement across the three datasets.

**Table 5:**
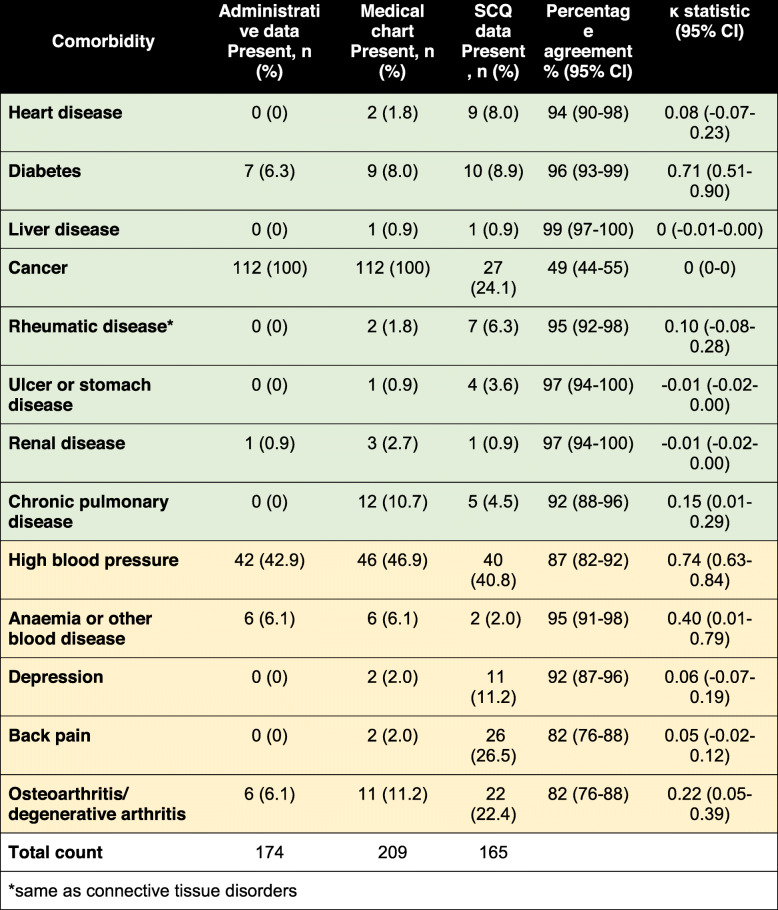
Comparison of comorbidities recorded in administrative data vs SCQ data vs medical charts (*n* = 112 for all six studied sites (green), *n* = 98 for all sites asides from Site 1 (yellow))

*same as connective tissue disorders

## Discussion

This study examined the concordance of comorbidity data extracted from administrative data, medical charts and self-reported. Our findings showed that agreement between comorbidity data collected by the Victorian Admitted Episodic Dataset, medical charts, and self-reports by men with CaP who have undergone a radical prostatectomy varied across the analysed comorbidities.

In terms of the comparison of all three data sources, a “slight”, less than “moderate” agreement was observed for most comorbidities when calculated using the kappa. The raw data for this study was also analysed to gain a more holistic understanding of concordance. No studies have previously compared these three data sources. However, studies have assessed concordance between two of these three groups. Six studies have analysed comorbidity concordance between patient self-reports and administrative data [16–21].. As with our study, most found high level of concordance for diabetes (four of six studies had kappa scores ranging from 0.70 and 0.83) [16–18, 20]. There was a variable level of agreement found for both myocardial infarct (kappa values between 0.14 and 0.75) [17–19] and asthma (kappa values between 0.11 and 0.66) [17, 18, 20]. Two out of the six studies which compared concordance of heart disease demonstrated only fair concordance (kappa values of 0.36 and 0.38) [16, 20]. Five articles were identified comparing patient self-reported comorbidities and medical record review; both also found substantial concordance for diabetes (kappa of 0.76) and hypertension (kappa of 0.75) [22–26].

Cancer was one of the most interesting comorbidities studied, with only 26% of respondents self-reporting the condition. This resulted in poor concordance with both medical record review and administrative data. Our findings correlate with those of Katz et al., who analysed concordance of Charlson comorbidities between the medical chart and self-reports among 170 hospitalised patients with a range of illnesses. Using a questionnaire that included all comorbidities captured in the Charlson index, Katz et al. found only moderate level of concordance (kappa of 0.45 (95% CI, 0.28 to 0.62)) between the medical chart and self-reports in regard to the presence of cancer [25]. It is likely that men did not report having cancer due to the framing of the SCQ question that asked: “Do you have cancer?” Given that the selected cohort all included men with prostate cancer who had undergone surgery, it may be that they believed having the prostate gland removed meant they no longer had cancer. However, while for many men this is the case, 27–53% of men will develop recurrence up to 10 years after radical prostatectomy and 16–35% of patients receive second-line treatment within 5 years of surgery [27].

There was a three-fold difference in reporting of depression (6 vs 17 reports), a nine-fold difference in reporting of back pain (4 vs 38 reports) and a nearly two-fold difference in reporting of osteoarthritis between medical charts and self-reports. Studies have highlighted that these conditions, in particular depression, are under-diagnosed in hospitals, with physicians not actively investigating whether patients have the condition due to its non-acute nature [28]. This has been especially noted with back pain, a chronic but non-life threatening comorbidity [29]. Patients with osteoarthritis have been noted to view their condition as part of the ageing process [30], with 50% of patients with severe knee pain not reporting to their physician about it [31] and thus reducing their inclination to actively seek medical assistance for their condition. In a study of 2380 community-dwelling patients aged 55–85 years a comparison of medical charts and self-report data showed a kappa of 0.31 (95% CI: 0.27–0.35) for osteoarthritis, with 21.8% of patients stating that they were affected by this condition even though their medical chart did not support the claim [32]. Those with a mobility limitation were more likely to self-report their condition than those without this limitation (OR: 2.68, 95% CI 2.10–3.44) [32]. Ultimately, this highlights that patient- and physician- specific views towards certain comorbidities can influence their tendency of being recorded in the medical charts or self-reports.

A systematic review by Leal and Laupland published in 2010 identified 13 articles comparing comorbidities captured using administrative and medical record data [33]. The pooled specificity (medical record set as the reference) in the administrative data ranged between 97.4 and 99.9%, yet the sensitivity varied significantly, ranging from 12.5 to 81.5%, suggesting that the absence rather than presence of a condition was more accurately recorded in administrative datasets.

In this study, we found that there was strong concordance between the administrative comorbidity data and the other studied data sources based on the reporting statistics used. However, after a more nuanced and deeper investigation, agreement with regard to the reporting of comorbidities was modest, at best. With conditions such chronic pulmonary disease and rheumatic disease, cases were often recorded in the medical chart but not in the administrative datasets.

Nine of the eighteen comorbidities compared between the medical charts and administrative datasets had no cases identified in the administrative data source. Of these nine conditions, chronic pulmonary disease was recorded 26 times in the medical chart but was not coded in the administrative dataset. A similar observation was seen with rheumatic disease and cerebrovascular disease, where it was recorded in the medical chart of 9 patients but was not coded for in the administrative dataset. There may be a few reasons for this observed discrepancy. Several studies have identified that hospital coders prioritise the coding of symptomatic comorbidities over asymptomatic ones, due to the higher level of hospital funding associated with the former [34] or due to Australian guidelines dictating that “additional diagnoses can only be assigned if they affect patient care during admission” [35, 36]. The first point was somewhat proven in the case of chronic pulmonary disease in this study, as it is a condition that may only manifest depending on certain environmental and physiological stimuli [37]. The Australian guidelines surrounding coding comorbidities is interesting, given that in the Australian Modification of the ICD-10 AM codes, coders are provided with ample space (fifty slots) to state any secondary diagnoses of a particular patient [25]. Other studies have highlighted how the level of experience of the hospital coder can impact the accuracy of the ICD-10 AM codes [38]. Differences in coding practice between inexperienced and experienced coders have been shown to exist [21, 33, 34, 39].

The “substantial” agreement reported by the kappa for diabetes may be attributed to the highly scrutinized nature of this condition in clinical settings [26]. Clinical coders are required to document ICD codes for conditions which require health services resources [34]. The codes are used to assign a Diagnosis Related Group (DRG), which in turn translates to funding for the health service. For patients with Type I diabetes, blood sugar levels are usually required at least twice daily, and insulin must be administered, usually by nursing staff. Type II diabetes is a condition that is monitored strictly within hospitals [40].

This study has several strengths. This is the first time that comorbidities have been compared across medical charts, administrative data and patient self-reports. Given the increasing focus on the use of patient reported data, this study enhances our knowledge on the reliability and accuracy of such data. This is particularly important as self-reporting comorbidities is a cost-effective way of collecting comorbidity data compared to the extraction of data from medical charts or administrative datasets [41].

While the SCQ survey has been validated, with good test-retest reliability being reported [23, 42], this is the first time that it has been examined in a prostate cancer population. This is despite the fact that it has been recommended by ICHOM as the preferred method for collecting comorbidities in men with localised prostate cancer [43]. However, more research is required before we can use it to risk adjust health outcomes. While pre-operative administration of the SCQ will likely improve the likelihood that patients will self-report cancer, there remain other comorbidities such as heart disease, which were reported by patients but not documented in the other data sources.

This study has a number of limitations which will impact the interpretation of the findings. One relates to the potential discrepancy in interpretation of the definitions for each comorbidity. A list of ICD-10 AM codes pertaining to certain conditions such as heart disease, lung disease and kidney disease were used to identify whether a patient was regarded to have the condition or not. These same criteria were not used uniformly in the other data sources. Indeed, the SCQ purposely diluted the complexity of the comorbidity labels to allow the conditions to be understood by patients “without any prior medical knowledge” [12]. This likely impacted concordance. For example, in this analysis, ischaemic heart disease (IHD) was not classified as “heart disease” as the ICD-10 AM codes pertaining to the two Charlson comorbidities of heart disease (myocardial infarction and congestive heart failure) did not consider IHD. However, patients with the condition may have self-reported it as heart disease.

Non-response bias may have influenced our results, given that we only had a response rate of 55.3%. It is not possible to know whether the non-responders would differ systematically from responders in terms of their self-reported comorbidities. Another bias likely introduced into this study relates to recall bias as men may have had trouble recalling comorbidities.

The sample size for this study was relatively small (*N* = 217), preventing sub-group analysis, such as whether there were differences based on type of hospital (public/private), where documentation practices may differ. Also, more nuanced findings were unable to be revealed, as shown in the difference in CCI distributions for the administrative datasets for *n* = 112 (population whom had data across all three data sources) and *n* = 201 (sample who had data across administrative dataset and medical charts). Incomplete datasets also prevented more nuanced investigations into the concordance of data.

## Conclusions

This study has shown that there are limitations in the recording of comorbidities for each dataset that impacts the ability to generate comorbidities indices. Using the SCQ as a risk adjustment tool will likely over represent comorbidities when compared to the use of medical reports or administrative data. The Charlson score, initially developed to be calculated from data extracted from medical charts, is likely to under-represent the presence of comorbidities when derived using administrative codes. In terms of raw counts of comorbidities, there were 392 and 310 in the medical charts and administrative codes, respectively.

Given the relative dis-concordance of comorbidity data, it appears that computing comorbidity indices from a chosen data source may not accurately capture the true health profile of the patient. This is a significant barrier that requires further investigations to determine the prognostic capacity of comorbidities on PSM or PCSM, outcomes that are currently reported by PCOR-Vic.

Further work is required to ensure that comorbidity-specific information is accurately collected for eventual risk-modelling purposes in hospital and clinician benchmarking reports. In clinical practice, administrative records may be the preferred source to collect comorbidity information due to the coded and accessible nature of the data. However, referring to complementary sources to extract comorbidities may result in a more holistic picture of a patient’s comorbidity profile to be understood. Improvements to the SCQ can be made such that it includes non-medical jargon that is familiar to patients. Automated modes to collect medical chart information should be considered. Future studies should also ensure that more robust statistical measures are employed when reporting on the level of agreement.

## Supplementary information

**Additional file 1 Supplementary Table 1.** Quality indicators reported by the PCOR- VIC registry (adapted from [5]). **Supplementary Table 2.** Charlson Comorbidity Index (CCI) [11]. **Supplementary Table 3.** Self-report comorbidity questionnaire [12].

## Data Availability

The datasets generated and/or analysed during the current study are not publicly available due to confidentiality but may be partially available from the corresponding author on reasonable request.

## Abbreviations

ICD-10 AM: International Classification of Diseases-10th edition (Australian modified)
PCOR-Vic: Prostate Cancer Outcomes Registry-Victoria
SCQ: Self-reported comorbidity questionnaire
CaP: Prostate cancer
PCSM: Prostate cancer specific mortality
NCCN: National Comprehensive Cancer Network
PSA: Prostate specific antigen
ADT: Androgen deprivation therapy
PSM: Positive surgical margins
CCI: Charlson Comorbidity Index
mCCI: Modified weighted CCI
