# Supplementary material for: (Dis)concordance of comorbidity data and cancer status across administrative datasets, medical charts, and self-reports
Source: BMC Health Serv Res. 2020 Sep 11;20:858. doi: 10.1186/s12913-020-05713-5 (PMC7488579; doi:10.1186/s12913-020-05713-5)
Supplement: Supplementary file 1 — Additional file 1 Supplementary Table 1. Quality indicators reported by the PCOR- VIC registry (adapted from [5]). Supplementary Table 2. Charlson Comorbidity Index (CCI) [11]. Supplementary Table 3. Self-report comorbidity questionnaire [12]. [file 12913_2020_5713_MOESM1_ESM.docx]

Supplementary Table 1: Quality indicators reported by the PCOR- VIC registry (adapted from (5))

| **Type of indicator** | **Indicator definition** |
| --- | --- |
| **Structural indicators** | - Volume (number) of cases treated (by type of treatment) at each site |
| **Process indicators** | - Clear documentation of clinical T stage |
|  | - Percentage of men with advanced disease (high‐ and very high‐risk groups according to NCCN or Cancer of the Prostate Risk Assessment [CAPRA] 6–10) who were given brachytherapy |
|  | - Percentage of men with advanced disease (high‐ and very high‐risk groups according to NCCN or CAPRA 6–10) who receive adjuvant hormonal therapy after post radical radiotherapy |
|  | - Percentage of men with PSA level recorded post prostatectomy |
|  | - Percentage of men with high‐risk disease who were managed with active surveillance - Percentage of patients with low-risk disease who were managed with surgery when they were suitable for active surveillance |
|  | - Percentage of cases where positive margins were reported on the histopathology report stratified by NCCN and CAPRA risk categories (positive surgical margins- PSM) |
| **Outcome indicators** | - Mortality rate, risk adjusted for NCCN risk group |
|  | - Disease‐free survival at 24 months |
|  | - Case‐reported assessment of urinary, sexual and bowel function and bother at 12 and 24 months after diagnosis |

Supplementary Table 2: Charlson Comorbidity Index (CCI) (11)

| **Points** | **Charlson Comorbidity Index** | |
| --- | --- | --- |
| 1 | Myocardial infarct | |
|  | Congestive heart failure | |
|  | Chronic pulmonary disease | |
|  | Connective tissue disease | |
|  | Ulcer disease | |
|  | Mild liver disease | |
|  | Diabetes | |
|  | Peripheral vascular disease | |
|  | Cerebrovascular disease | |
|  | Dementia | |
| 2 | Moderate/severe renal disease | |
|  | Diabetes with end organ damage | |
|  | Hemiplegia | |
|  | Any tumour | Grouped together in the Deyo-modified CCI |
|  | Leukaemia |  |
|  | Lymphoma |  |
| 3 | Moderate/severe liver disease | |
| 6 | Metastatic solid tumour | |
|  | AIDS | |
| Note: Colour-coded conditions were captured across all three studied data sources | | |

Supplementary Table 3: Self-report comorbidity questionnaire (12)

| *ID NUMBER* | A. Do you have this problem? | | B. Do you receive treatment for it? | | C. Does it limit your activities? | |
| --- | --- | --- | --- | --- | --- | --- |
| PROBLEM | No | Yes | No | Yes | No | Yes |
| Heart disease | **o** | **o** | **o** | **o** | **o** | **o** |
| High blood pressure | **o** | **o** | **o** | **o** | **o** | **o** |
| Lung disease | **o** | **o** | **o** | **o** | **o** | **o** |
| Diabetes | **o** | **o** | **o** | **o** | **o** | **o** |
| Ulcer or stomach disease | **o** | **o** | **o** | **o** | **o** | **o** |
| Kidney disease | **o** | **o** | **o** | **o** | **o** | **o** |
| Liver disease | **o** | **o** | **o** | **o** | **o** | **o** |
| Anemia or other blood disease | **o** | **o** | **o** | **o** | **o** | **o** |
| Cancer | **o** | **o** | **o** | **o** | **o** | **o** |
| Depression | **o** | **o** | **o** | **o** | **o** | **o** |
| Osteoarthritis or degenerative arthritis | **o** | **o** | **o** | **o** | **o** | **o** |
| Back pain | **o** | **o** | **o** | **o** | **o** | **o** |
| Rheumatoid arthritis | **o** | **o** | **o** | **o** | **o** | **o** |
| Other medical problem, please specify:  …………………………………………………. | **o** | **o** | **o** | **o** | **o** | **o** |
| Other medical problem, please specify:  …………………………………………………. | **o** | **o** | **o** | **o** | **o** | **o** |
